# Mnemonic Nomenclature

**Published:** 1836

**Authors:** John L. Riddell


ART. III. — MNEMONIC NOMENCLATURE,—*Applied to the Proximate, Organic Principles.* By JOHN L. RIDDELL, M. D.

The systematic method pursued in giving names to mineral compounds, so beautiful in theory, and so admirable and instructive in practice, can never be extended to the chemistry of animals and plants. Rising above the simplicity which characterizes the spontaneous combinations of lifeless elements, the organic kingdoms present us with numberless substances, marked with diversity in appearance, consistence and mechanical properties; which, though in these particulars they seemingly exhaust the endless changes of variety, are yet constituted from only three or four distinct elements of matter. The composition of most proximate principles, though definite like that of the simplest mineral, is necessarily too complex to be expressed by the ordinary mode of naming.

The factitious names in the following table, indicate not only the kinds of ultimate elements, but the precise ratio in which they are combined. Selecting the more important compounds, as those to which a star (*) is prefixed, a very short time only will be required, to associate and fix indelibly in the memory, the proposed and common names;—an acquisition which chemists, physicians, apothecaries and students in physiology, will doubtless know how to appreciate.

Since devising and applying this method of notation, I have observed that Dr. Abercrombie, in his "Enquiries concerning the intellectual powers," makes mention of the mnemonic system of M. Feinagle, for remembering dates; which is founded on a plan somewhat similar.

| *Elements.* | *Symbols.* | *Atomic weights, or combining proportions.* |
|---|---|---|
| Oxygen - - | *o* - - | 8 |
| Hydrogen - - | *e* - - | 1 |
| Nitrogen - - | *a* - - | 14.15 |
| Carbon - - | *u* - - | 6.12 |

*System of Numerals.*

| | | | | | |
|---|---|---|---|---|---|
| *d* represents | 1 | | *s* | — | 7 |
| *l* — | 2 | | *t* | — | 8 |
| *r* — | 3 | | *z* | — | 9 |
| *m* — | 4 | | *i* | — | 10 |
| *v* — | 5 | | *ii* | — | 100. |
| *p* — | 6 | | | | |

Rule 1. In the formation of a name, those numeral and elementary symbols referring to each other are placed side by side, the former on the left hand, the latter on the right.

Rule 2. When a numeral consonant occurs on the left hand of the numeral vowel *i*, the two letters are factors ;— they are to be multiplied together. When the consonant occurs on the right hand, its value is to be added to that of the vowel.

*Examples.* *mi* = 40. *im* =14. *mim* = 44. *tip* = 86. *Luro*—(oxalic acid) = two equivalents of carbon and three of oxygen.

Note. The letter *d*, when a factor, may either be prefixed or omitted, as the harmony of the syllables require.

It will be observed that the factitious names representing acids, terminate in *o* ; those representing alkalies in *a* ; inflammables in *u* ; and most neutral bodies in *e*.

The enumeration which follows, comprises all those proximate principles, of which satisfactory analyses have yet been published. The composition indicated, will be found to correspond either perfectly or closely, with the analytical results adopted in the fifth edition of Turner's Chemistry.

### CLASS I.—*Acids.*

### § 1. ACIDS CONSISTING OF OXYGEN AND CARBON.

*Luro*—oxalic acid
*Muro*—mellitic
*Vumo*—croconic
*Vupo*—amylic acid.

### § 2. ACIDS COMPOSED OF OXYGEN, CARBON AND HYDROGEN.

* *Remuro*—acetic acid
*Mepumo*—lactic
*Mulemo*—malic
*Luero*—formic
*Mulemo*—citric
*Diulero*—pyrocitric
*Mulevo*—tartaric
*Mulevo*—racemic
*Surevo*—gallic
*Repuro*—pyrogallic
*Reiluro*—metagallic
*Sulemo*—ellagic
*Dieivudio*—kinic
*Suleso*—meconic
*Ilumedio*—metameconic
*Ituzeto*—catechucic
*Mulero*—succinic
*Puveto*—mucic
*Diuzero*—valerianic
*Ipudipemo*—rocellic
*Liudipevo*—camphoric
*Imuvero*—benzoic
*Siupisevo*—stearic
*Siupivepo*—margaric
*Siuvitevo*—oleic
*Sediuro*—phocenic acid.

§ 3. ACIDS CONTAINING NITROGEN.

*Lavulero*—uric acid
*Aliuzepo*—hippuric
*Avetupo*—aspartic
*Lavumemo*—alantoic
*Ramivuiverio*—indigotic
*Repuraro*—cyanuric
*Ivurairo*—carbazotic
*Aludo*—cyanic (not organic)
*Alude*—hydrocyanic acid.

### CLASS II.—*Vegetable Alkalies.*

*Porimuitea*—morphia
*Isovimulisea*—narcotina
*Voriulidea*—codeia
*Imolulidea*—narceia
*Oliudilea*—cinchonia
**Loliudilea*—quinia
*Roliudilea*—aricina
*Roriudipea*—strychnia
*Poriluditea*—brucia
*Porimulilea*—veratria
*Zorivulipea*—emetia
*Loriulidea*—delphia.

### CLASS III.—*Inflammables.*

§ 1. ETHEROID SUBSTANCES.

**Orelu*—alcohol
*Ovemu*—ether
*Movepu*—oxalic ether
*Moideitu*—benzoic ether
*Moletu*—acetic ether
*Posetu*—tartaric ether
*Lovemu*—pyroxylic spirit
*Oreru*—pyro-acetic spirit.

§ 2. RESINOID BODIES.

*Loideimu*—common resin
*Lirelitu*—essence of turpentine
*Voireliu*—oil of cloves
*Loveimu*—benzule
*Lopeimu*—oil of bitter almonds
*Oteidu*—caryophylline
*Otediu*—camphor
*Oiediu*—camphor from oil of peppermint
*Opediu*—camphor from oil of anise.

§ 3. CEROID BODIES.

*Siserotilu*—cetine
*Sizesopizu*—spermaceti
*Isedoipu*—ethal
*Vizelosidu*—cholesterine
*Pirelopizu*—ambreine
*Pitetosizu*—human fat
*Pitetositu*—oleine of human fat
*Pisetosizu*—hogslard
*Pitesosizu*—oleine of hogslard
*Siesosiru*—stearine of mutton suet
*Pipesosizu*—oleine of mutton suet
*Romieviru*—olive oil
*Oideiru*—beeswax.

### CLASS IV.—*Neutral Bodies.*

§ 1. PRINCIPLES CONSISTING OF OXYGEN, HYDROGEN AND CARBON.

**Tozute*—cane sugar
*Posupe*—wheat starch
*Iloivudire*—potato starch
*Pitopivupite*—mannite
*Iroimudime*—gum Arabic
**Idoisudile*—lignin
*Morume*—ulmin
*Moriulime*—piperin
*Olule*—salicin
*Mozume*—meconin
*Zoidumire*—elatin
*Rorume*—glycerine.

§ 2. AZOTIZED PRINCIPLES.

*Momivuivera*—indigogen
*Pomivuivera*—indigo blue
*Potetula*—asparagin
*Lotuvela*—caffein
*Loseimuda*—benzamide
**Ruperora*—urea
*Zeroizura*—cystic oxide
*Ivuidelola*—caseum
**Rivulitedivora*—fibrin
*Rilurilediinora*—gelatine
*Rirurideiroma*—albumen.

### *Inorganic binary compounds of the same elements, not embraced in the foregoing classification.*

COMPOUNDS OF OXYGEN AND HYDROGEN.

*Ode*—water
*Lode*—peroxide of hydrogen.

COMPOUNDS OF OXYGEN AND NITROGEN.

§ 1. *Neutral bodies.*

*Oda*—nitrous oxide

*Loda*—nitric oxide.

§ 2. *Acids.*

*Aro*—hyponitrous acid
*Amo*—nitrous acid
*Avo*—nitric acid.

COMPOUNDS OF OXYGEN AND CARBON.

§ 1. *Inflammable.*

*Odu*—carbonic oxide.

§ 2. *Acid.*

**Ulo*—carbonic acid.

COMPOUND OF HYDROGEN AND NITROGEN.

*Rea*—ammonia.

COMPOUND OF HYDROGEN AND CARBON.

*Leu*—light carburetted hydrogen

*Lelu*—olefiant gas

*Repu*—bicarburet of hydrogen

*Memu*—etherine

*Vemu*—ethule

*Vepu*—naptha

*Mediu*—napthaline

*Pedivu*—paranapthaline

*Tediu*—camphene

*Mevu*—citrene.

